# Leaf herbivory by insects during summer reduces overwinter browsing by moose

**DOI:** 10.1186/s12898-018-0192-x

**Published:** 2018-09-27

**Authors:** Brian P. Allman, Knut Kielland, Diane Wagner

**Affiliations:** Institute of Arctic Biology, Department of Biology and Wildlife, University of Alaska Fairbanks, Fairbanks, Alaska USA

**Keywords:** Indirect effects, Herbivory, Browsing, Leaf mining, Defoliation, Herbivory, Insect outbreak, *Salix interior*, *Micrurapteryx salicifoliella*

## Abstract

**Background:**

Damage to plants by herbivores potentially affects the quality and quantity of the plant tissue available to other herbivore taxa that utilize the same host plants at a later time. This study addresses the indirect effects of insect herbivores on mammalian browsers, a particularly poorly-understood class of interactions. Working in the Alaskan boreal forest, we investigated the indirect effects of insect damage to *Salix interior* leaves during the growing season on the consumption of browse by moose during winter, and on quantity and quality of browse production.

**Results:**

Treatment with insecticide reduced leaf mining damage by the willow leaf blotch miner, *Micrurapteryx salicifoliella,* and increased both the biomass and proportion of the total production of woody tissue browsed by moose. *Salix interior* plants with experimentally-reduced insect damage produced significantly more stem biomass than controls, but did not differ in stem quality as indicated by nitrogen concentration or protein precipitation capacity, an assay of the protein-binding activity of tannins.

**Conclusions:**

Insect herbivory on *Salix*, including the outbreak herbivore *M. salicifoliella*, affected the feeding behavior of moose. The results demonstrate that even moderate levels of leaf damage by insects can have surprisingly strong impacts on stem production and influence the foraging behavior of distantly related taxa browsing on woody tissue months after leaves have dropped.

**Electronic supplementary material:**

The online version of this article (10.1186/s12898-018-0192-x) contains supplementary material, which is available to authorized users.

## Background

Herbivory can alter aspects of plant phenotype, including growth, defense, and nutritional composition [1, 2], and such changes can in turn affect the behavior and performance of herbivore taxa that feed on the same host plant at a later point in time [3, 4]. These indirect effects of one herbivore taxon on another, mediated by changes in host plant density and plant trait expression, are common and increasingly recognized as important forces in shaping community organization [3, 5, 6].

Indirect interactions may occur between distantly-related herbivore taxa that differ greatly in body size and feed on different plant tissues at different times of year, such as insect folivores and mammalian browsers [7]. Browsing can have strong effects on plant performance, species composition, and vegetation structure [8–10], and the impacts of browsers on plant performance and mortality typically exceed those of insects [7, 11–15]. It is therefore not surprising that browsers also tend to have strong direct and indirect effects on herbivorous insects, a hypothesis addressed by numerous studies and several recent reviews [7, 16, 17]. In contrast, the effects of insect herbivory on mammalian browsing have rarely been studied and remain a poorly understood aspect of interaction ecology [7]. Damage to leaves caused by insect feeding could impact the foraging behavior of browsers by reducing plant quantity and quality [18]. Leaf damage by insects can decrease the size of shoots on shared host plants [19–22], reducing forage availability for browsers and potentially altering patterns of food selection by browsers that prefer host species and individuals with large stems over small [23, 24]. Leaf damage may also influence subsequent browse quality by altering the nutritional and defensive chemistry of stems. For example, heavy defoliation of aspen increased the concentration of nitrogen and reduced the concentration of phenolic glycosides and condensed tannins in aspen stems [25]. Regardless of mechanism, a change in food selection by browsers in response to prior insect herbivory could potentially modify the direct fitness effects of insect herbivory on plants.

The potential for insect herbivores to affect mammalian browsers is particularly relevant in the Alaskan boreal forest, where large-scale insect outbreaks are common and browsers include ecologically and economically important wildlife species such as moose and snowshoe hares. During the past 20 years, a persistent long-term outbreak of the aspen leaf miner, *Phyllocnistis populiella* Chambers, and recurrent outbreaks of the willow leaf blotch miner, *Micrurapteryx salicifoliella* (Chambers 1872), have caused widespread damage to *Populus* and *Salix* spp., respectively [26–28]. These genera, and *Salix* in particular, provide a critical source of forage for vertebrate browsers [29, 30], comprising from 43% [31, 32] to over 90% of moose winter diet [32]. When high levels of folivory occur on preferred browse species, insect damage could potentially affect forage availability for, and host plant selection by, moose and other browsers.

During an outbreak of the leaf miner *M. salicifoliella*, we investigated whether insect folivory during the growing season altered the consumption of, and preference for, winter-dormant stems of *Salix interior* Rowlee by moose. We hypothesized that insect folivory would reduce stem production, resulting in lower consumption of, and preference for, willow stems on unmanipulated study subplots relative to paired subplots with experimentally-reduced levels of insect folivory. In addition, we investigated whether insect folivory altered tannin protein precipitation capacity [33] and stem nitrogen concentration.

## Results

### Patterns of herbivory

**Fig. 1:**
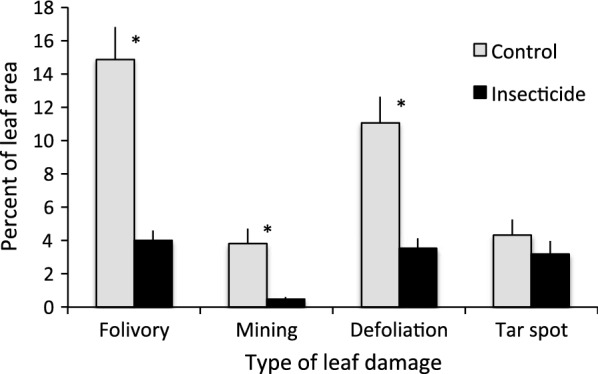
Patterns of leaf damage sustained by *S. interior* plants that received natural levels of herbivory (controls) and plants treated with insecticide to reduce folivory. Types of damage shown are total folivory (the sum of all insect feeding damage), leaf mining alone, missing tissue removed by external leaf chewers and skeletonizers alone, and infection by tar spot fungus (*Rhytisma acerinum*). Asterisks identify statistical significance at *P *< 0.05

Treatment with insecticide successfully reduced foliar damage caused by insects (Fig. 1). Average percent folivory, calculated as the sum of leaf mining and external damage to leaf tissue, was 3.7-fold lower on plants in insecticide-sprayed subplots than on control subplots, a statistically significant difference (Fig. 1; *F *= 20.35, *df* = 1, 5.1, *P *= 0.006). When considered separately, damage caused by the leaf miner *M. salicifoliella* and externally-feeding insects were not equally affected by insecticide treatment (Fig. 1). Treatment reduced leaf mining strongly, by 7.9-fold relative to control (*F* = 47.53, *df* = 1, 4.1, *P *= 0.002), but reduced damage by externally-feeding herbivores by only 3.1-fold (Fig. 1; *F* = 5.99, *df* = 1, 5.1, *P *= 0.06). We found little evidence of non-target effects caused by the insecticide treatment. The percent of leaf area affected by tar spot fungus was slightly, but not significantly, reduced by insecticide treatment (Fig. 1; *F* = 3.80, *df* = 1, 5.1, *P *= 0.11).

Moose tend to feed on highly digestible, high-quality foliage during summer, foregoing the consumption of lignified stems until after the leaves have fallen in autumn [34–36]. In the vicinity of the Tanana River, it is rare for moose foraging in summer to consume riverbank willows such as *S. interior*, which has small, thick leaves, instead feeding in habitats with better-quality forage (Kielland, personal observation). As expected, we noted no evidence of leaf-stripping or recent stem browsing of *S. interior* on the study plots during our summer surveys of leaf damage.

### Browse quantity and production

**Fig. 2:**
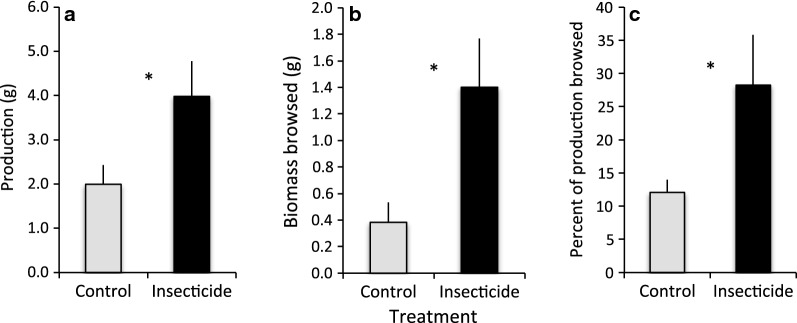
Effect of insecticide treatment during the growing season on subsequent stem production by, and overwinter browsing on, *Salix interior*: panel **a** stem biomass produced, panel **b** stem biomass browsed; panel **c** proportion of stem production browsed. Means and standard errors (g per plant) are based on a sample size of 35 plants each for treatment and control. Asterisks identify statistical significance at *P *< 0.05

Experimental reduction of insect folivory increased stem production and overwinter browsing intensity. On average, *S. interior* plants on insecticide-treated subplots produced twice as much stem biomass as controls (Fig. 2a; F = 11.07, *df* = 1, 4.9, *P* = 0.02). The biomass removed by browsers over winter was 3.7-fold greater on insecticide-treated subplots than on control subplots (Fig. 2b, *F* = 12.36, *df* = 1, 4.7, *P* = 0.02), and browsed biomass constituted a significantly (2.3-fold) greater percentage of production on insecticide-treated subplots than controls (Fig. 2c; *F* = 18.49, *df* = 1, 4.0, *P* = 0.01). Based on the appearance of browse scars, the vast majority of browse on the study plots was consumed by moose, which is consistent with the very low hare densities detected during the study [37].

### Browse quality

In contrast to browse quantity, there was no evidence that treatment with insecticide altered the nutritional quality of *S. interior* stem tissue. Nitrogen concentration of current annual stems averaged 1.10% (SD = 0.27, n = 60 plants) and did not differ between insecticide-sprayed and control subplots (sprayed: 1.05% ± 0.04 SE, n = 29; control: 1.15% ± 0.05 SE, n = 31; *F* = 1.11, *df* = 1, 4.9, *P* = 0.34). Likewise, average protein precipitation capacity also did not differ between sprayed and control subplots (*F* = 0.09, *df* = 1, 4.9, *P* = 0.77), averaging 411 mg/g ± 25 SE (n = 29) and 390 mg/g ± 20 SE (n = 31) for plants on treatment and control subplots, respectively.

## Discussion

Experimental reduction of folivory during the growing season altered patterns of moose browsing on *S. interior* the following winter. Moose removed more stem biomass, and a greater proportion of the biomass produced, from plants growing on subplots treated with insecticide the previous spring than from plants subject to ambient levels of insect damage. The results suggest that moose preferred to consume plants that had sustained lower levels of leaf damage during the growing season, and consequently had higher stem biomass. Although experimental artifacts are always possible, we find it unlikely that moose responded directly to the presence of insecticide residues. Plants were treated in spring but browsing occurred many months later, in fall and winter. Spinosad, the insecticide used in this experiment, has a half-life of only 1.6–16 days on plant tissues [38] and in addition was likely rinsed from stems by rain. Moreover, if insecticide residue did remain on stems it would more likely discourage browsing than encourage it. The results are better explained as a response by browsers to changes in plant characteristics related to folivory. Ours is one of few studies to investigate the plant-mediated effects of insect herbivory on browsing. The results are qualitatively similar to those of Strauss [18], who reported that beetle damage to sumac (*Rhus glabra*) reduced the likelihood of subsequent browsing by deer. In contrast, Gómez and González-Megías [39] found no effect of beetle herbivory on the interaction between sheep browsers and the shared host plant, *Hormathophylla spinosa*.

The plant characteristics to which moose responded were more likely associated with quantity than quality. Several previous studies suggest that moose prefer stems and plants with more biomass [23, 40], and increased browse availability can result in disproportionate increases in forage consumption by moose [41]. A preference for plants with high biomass production, perhaps motivated by feeding efficiency due to greater stem densities, could explain the pattern of browsing detected in this study. This and a previous study conducted at other sites within interior Alaska indicate that leaf mining by *M. salicifoliella* can have strong negative effects on the stem production of susceptible *Salix* species [42]. In the current investigation, ambient levels of leaf damage averaged only 15% of leaf area (Fig. 1), yet experimental reduction of folivory to 4% doubled stem biomass production (Fig. 2). Our estimates of leaf mining might, however, have underestimated actual damage to the interior of affected leaves, because we quantified only the damage visible on the leaf surface. Because we estimated production on unfenced plots, we cannot dismiss the possibility that compensatory growth in response to moose browsing during the growing season also influenced stem biomass. However, two lines of evidence suggest this is unlikely. First, it is rare for moose to browse on willow stems or leaves along the bank of the Tanana River during the growing season, when higher quality vegetation is abundant elsewhere, and as expected we noted no damage characteristic of moose during leaf surveys in July and August. Second, whereas some willows can compensate for herbivory [43, 44], the browsing intensity in our study system is of such a magnitude (circa 30%) as to largely negate such a response [45].

The chemical characteristics of plants, such as concentrations of defensive compounds or protein, can also affect the feeding behaviors of browsers [30, 46], but in this case we found no evidence that a change in browse quality in response to folivory was responsible for the observed pattern of browsing. Neither the concentration of nitrogen nor the protein precipitation capacity of *S. interior* stem tissues varied significantly between treatment and controls. The ability to detect differences driven by treatment is limited by aspects of our study design; specifically, there were a small number of experimental units (six pairs of subplots) and no pre-treatment measurements of plant quality. However, it is clear that quantitative responses by plants greatly exceeded qualitative responses for the aspects of plant composition measured.

Our experimental treatment was more effective at reducing leaf mining damage by *M. salicifoliella* than by externally-feeding herbivores, likely because the single application of insecticide in spring was timed to have maximum impact on the early instars of the leaf miner. The larger impact of treatment on leaf miners relative to other herbivores suggests that *M. salicifoliella* was largely responsible for the reduction in plant growth on control subplots, although we cannot dismiss the potential effect of external leaf feeders. Since first documented in interior Alaska in 1991 [26, 47], *M. salicifoliella* has caused widespread damage to multiple *Salix* species during recurrent outbreaks [48]. During some outbreak years, average leaf mining damage to *S. interior* and other *Salix* species have been higher than levels recorded in this study [42].

## Conclusions

The results of this field experiment show that even modest levels of insect folivory can reduce moose browsing both absolutely and relative to plant production. The most likely mechanism for this pattern is a preference by moose for plants with relatively high stem production. We conclude that insect herbivory can impact the behavior of vertebrate taxa feeding on different plant tissues months after the growing season. In light of the predicted increases in the frequency and intensity of insect outbreaks [49–51], indirect interactions among herbivore taxa may have stronger ecological consequences as the climate continues to warm.

## Methods

### Natural history

Sandbar willow, *S. interior*, is an erect shrub common to disturbed riverine sites in interior Alaska. It colonizes newly-deposited alluvium and tends to propagate vegetatively by producing shoots from underground roots [52]. The leaves of *S. interior* are glabrous and contain condensed tannins but not phenolic glycosides [53]. In parts of interior Alaska, including the Tanana River area where this study occurred, it is a preferred source of moose browse during winter [54]. During the growing season, *S. interior* is attacked by a range of externally-feeding insect defoliators, including chrysomelid beetles and Lepidoptera, and by the internally-feeding leaf miner *M. salicifoliella*. *Micrurapteryx salicifoliella* is a gracillariid moth that attacks a wide range of *Salix* species [55]. Though native to North America, *M. salicifoliella* was only first documented in Alaska in the early 1990s [26]. Larvae damage plants by feeding on mesophyll and by breaching the foliar cuticle, causing desiccation [42].

### Study area

The study was conducted during 2012 and 2013 within the Bonanza Creek Long Term Ecological Research area on early-successional floodplain habitat along the Tanana River, approximately 20 km southwest of Fairbanks, Alaska. The vegetation was dominated by *Salix* species, with low densities of balsam poplar (*Populus balsamifera*), and alder (*Alnus tenuifolia)*. Location and vegetation composition of study plots is provided in Additional file 1.

### Experimental design

The aim of the study was to test whether suppression of insect herbivory during the growing season affected browse production and consumption over winter. We established two 9 × 12 m plots on each of the six study sites during May 2012. One plot within each pair, chosen at random, was surrounded by a 9 m × 12 m × 2 m tall metal chain-link fence. Each fenced and unfenced plot was divided into two subplots measuring 4 × 7 m with a 1 m buffer strip on all sides. Subplots within plots were randomly assigned to insecticide-suppression treatment or control. In the context of this study, the mammal-exclusion plots were used to assess the effects of insect folivory on plant quality in the absence of mammal browsing, while the unfenced plots were used to assess the effect of insect suppression on production and browsing.

All shrubs within subplots assigned to the insect suppression treatment were sprayed with insecticide during the first week of June 2012, when oviposition by *M. salicifoliella* had slowed but larvae were still in early instars. We used the insecticide Spinosad (Conserve SC, EPA registration number 62719-291; Dow AgroSciences, Indianapolis, Ind.; 1.56 mL/L, plants sprayed to runoff), which is effective against both leaf miners and externally-feeding insect herbivores, has a rapid rate of degradation when exposed to light, and is a relatively low threat to mammalian health [38]. All shrubs within control subplots were sprayed with an equivalent volume of water.

### Patterns of herbivory

In order to test the effectiveness of the insecticide treatment, we quantified leaf damage on insecticide-treated and control subplots in late summer 2012. We chose at random 5–6 individuals of *S. interior* on each subplot and assessed leaf damage on each leaf of two haphazardly-chosen shoots per plant. Leaf damage was estimated visually and non-destructively as the percentage leaf surface area mined and the area removed or skeletonized by externally feeding insects. All observers were tested prior to collecting data by comparing their visual estimates of leaf damage on three *Salix* species (20–30 leaves per species) to measurements of the same leaves made using image analysis software (Image J, National Institutes of Health, Bethesda, Maryland). Visual estimates of damage were strongly related to digital measurements (*R*^2^ ≥ 0.95 for all observers). The only leaf mining present was characteristic of the damage caused by *M salicifoliella*. Many *S. interior* plants were also infected by tar spot fungus (*Rhytisma acerinum*). We estimated the percent of leaf area affected by the fungus in order to assess non-target effects of the insecticide.

### Browse quantity and production

During April and May 2013, we measured both the production of current annual growth and the biomass of woody tissue browsed over winter for 5–6 randomly-selected plants at least 35 cm in height on each subplot (mean height 63 cm, SD = 18, n = 118). For each plant, we recorded the total number of current annual growth stems, the diameter at the base of 10 current annual growth stems per plant (DCAG), and the diameter at the point of browsing (DPB) for a total of an up to 10 browsed stems if browsing was present. We used the DCAG to predict production and DPB to predict browsing by applying species-specific equations relating stem diameter to biomass, following Paragi et al. [56]. The equations we applied were developed from measurements of *S. interior* plants sampled near the study sites (n = 71). Total production per plant was calculated as the product of the number of current annual growth stems and the average stem biomass, and total browse per plant was calculated as the product of the number of browsed stems and the average browsed stem biomass.

### Browse quality

To investigate the effect of insect folivory on the quality of woody tissue for browsers, we measured the effect of insect suppression on N content and protein precipitation capacity, a measure of tannin activity. Samples were collected from the insecticide-treated and control subplots within mammal exclosures only, in order to avoid confounding the effects of insect and mammalian herbivory. We harvested three CAG stems from each of five randomly selected plants on each subplot. Only stems DCAG ≤ 2 mm were collected, in order to minimize variation in stem chemistry related to size. Stem samples were transported on ice to the University of Alaska Fairbanks and stored at − 80 °C. Samples were lyophilized for 48–72 h, pooled within plants, and ground using a Wiley mill over a 40-mesh screen. N composition as a percentage of dry mass was measured in duplicate 0.1 g subsamples using a LECO 2000 CNS Analyzer (LECO Instruments, St. Joseph, MI, USA).

Protein precipitation capacity was measured using the method of Robbins et al. [57] modified for a micro-plate reader. Samples of 0.50 g were soaked in 20.0 mL of 50% methanol for 5 min, sonicated, and allowed to incubate at room temperature for 30 min. The solution was centrifuged for 15 min at 5000 RPM. 35.0 µL of the supernatant was combined with 140.0 µL of 5 mg/mL bovine serum albumin (BSA) in 0.2 M acetic acid acetate buffer and 0.17 M NaCl within a 96-well centrifugable microplate cell. Microplates were centrifuged for 10 min at 6000 RPM, after which 5.0 µL of the supernatant was combined with 250.0 µL of Bio-Rad Quick Start Bradford Protein Assay Reagent (Bio-Rad Laboratories, Hercules, CA, USA) and incubated for 6 min. Absorbance was read at 590 nm and protein content determined with reference to a standard curve. We calculated BSA precipitated by subtracting the soluble protein mass from the initial mass of BSA added to each well. Protein precipitation capacity was calculated by dividing the mass of BSA precipitated by the original mass of ground sample in each well, and is reported as mg BSA precipitated per g dry sample.

### Data analysis

All dependent variables were averaged within plants and analyzed using general linear mixed models. Data were log or log(x + 1) transformed when necessary to meet model assumptions. Analyses were conducted using JMP 13 software (SAS Institute, Cary, NC). All dependent variables were modeled separately as the fixed effect of insecticide treatment and the random effects of plot, to account for the pairing of subplots within plots, and treatment nested within plot, to account for the non-independence of plants within subplots.

## Additional file

**Additional file 1.** Location and shrub composition of experimental plots.
