# Supplementary material for: Leaf herbivory by insects during summer reduces overwinter browsing by moose
Source: BMC Ecol. 2018 Sep 27;18:38. doi: 10.1186/s12898-018-0192-x (PMC6161349; doi:10.1186/s12898-018-0192-x)
Supplement: Supplementary file 1 — Additional file 1. Location and shrub composition of experimental plots. [file 12898_2018_192_MOESM1_ESM.docx]

Additional File 1. Location and 2012 shrub composition of experimental plots.

| **Site Coordinates** | | | **% Ground Cover by Species** | | | | | |
| --- | --- | --- | --- | --- | --- | --- | --- | --- |
| Plot | Latitude | Longitude | *S. inte* | *S. niph* | *S. pseu* | *S. alax* | *P. bals* | *A. tenu* |
| 1 | 64 42.47° N | 148 09.15° W | 7.6 | 0.3 | 3.5 | 0.0 | 2.6 | 0.4 |
| 2 | 64 42.51° N | 148 09.12° W | 9.5 | 1.6 | 0.9 | 0.0 | 0.6 | 0.5 |
| 3 | 64 42.50° N | 148 10.43° W | 5.3 | 11.2 | 0.3 | 0.2 | 0.2 | 0.1 |
| 4 | 64 42.38° N | 148 13.16° W | 11.0 | 2.3 | 0.4 | 0.0 | 1.6 | 0.2 |
| 5 | 64 40.53° N | 148 17.86° W | 6.4 | 5.7 | 2.8 | 0.6 | 1.1 | 1.5 |
| 6 | 64 40.54° N | 148 17.41° W | 2.9 | 2.9 | 3.9 | 0.0 | 0.9 | 0.0 |

Species names are coded as follows: *S. niph = Salix niphoclada, S. inte = S. interior, S. pseu = S. pseudomyrsinites, S. alax = S. alaxensis, P. bals = Populus balsamifera, A. tenu = Alnus tenuifolia.*
